# Social and demographic characteristics of a Polish cohort with Wilson disease and the impact of treatment persistence

**DOI:** 10.1186/s13023-019-1133-2

**Published:** 2019-07-05

**Authors:** Wojciech Maselbas, Tomasz Litwin, Anna Czlonkowska

**Affiliations:** 1 Department of Experimental and Clinical Pharmacology, Medical University of Warsaw, Warsaw, Poland; 2 2nd Department of Neurology, Institute of Psychiatry and Neurology, Warsaw, Poland

**Keywords:** Wilson disease, Neurological manifestations, Treatment persistence

## Abstract

**Background:**

Wilson disease (WD) is a genetic disorder involving impaired copper metabolism, which presents with hepatic, neurological, and/or psychiatric manifestations. WD requires lifelong pharmacotherapy and treatment persistence may be problematic. We studied social characteristics, education, and work-related activities and how they are affected by WD symptoms and treatment persistence.

**Methods:**

In a cross-sectional study, data on demographic characteristics, achieved education level, household and marital status, plus a primary source of income were collected from 202 Polish subjects (mean ± standard deviation age of 36.4 ± 9.9 years at assessment) with WD.

**Results:**

Overall, WD appeared to have a negative impact on achieved level of education and influenced the ability to work as compared with the general Polish population. Patients with neurological manifestations less often achieved upper-secondary/post-secondary or higher education compared with those with hepatic manifestations (65.5% vs. 83.6%; *p* = 0.003). They also significantly less frequently stated salary (19.6% vs. 56.2%; *p* < 0.0001) as the primary income and more often were on disability pension (53.3% vs. 26.0%; *p* = 0.0003). The percentage of married patients with WD appeared lower than in the general population (47.0% vs. 54.6%), although the difference was not significant (*p* = 0.2). The 27.6% of patients who were non-persistent with WD treatment less frequently achieved upper/post-secondary or higher education compared with persistent patients (66.0% vs. 76.3%; NS) and their primary source of outcome was significantly less often a salary (18.9% vs. 40.3%; *p* = 0.001).

**Conclusions:**

Neurological manifestations had an adverse effect on education level and work ability. Treatment non-persistence had a further negative impact regardless of the disease form. Patients with WD should receive appropriate treatment, with the need for persistence emphasized and monitored to avoid a detrimental effect on their lives.

## Introduction

Wilson disease (WD) is an autosomal recessive disorder of impaired copper metabolism caused by mutations in the *ATP7B* gene, which encodes a copper-transporting ATPase involved in copper transportation across cell membranes [1]. Decreased ATP7B function leads to reduced incorporation of copper into ceruloplasmin and impaired biliary excretion. The general prevalence of WD is thought to be around 1 in 30,000 people but prevalence varies among populations and WD may be underrecognized and underdiagnosed [2, 3].

The onset of disease is mainly in children and younger adults, but it is not infrequent to find patients who have their first symptoms during adulthood. The clinical symptoms of WD are initially related to accumulation of copper in the liver, which often leads to hepatic insufficiency. In most patients, hepatic copper accumulation is followed by toxic effects on the nervous system, especially in the brain. If untreated, WD frequently leads to severe impairment of normal functions and death within several years from development of symptoms [4].

WD is one of the few inborn metabolic diseases that can be successfully treated [5]. Pharmacotherapy is based on agents that eliminate copper from the body via urinary excretion (d-penicillamine or trientine) or prevent copper absorption via the gastrointestinal tract (zinc salts). In the majority of cases, early diagnosis and lifelong treatment help to prevent irreversible damage [6]. Unfortunately, in some patients, the disease is relentlessly progressive despite treatment [7]. Furthermore, treatment persistence can be problematic for some patients with WD [8]; failure to take lifelong therapy may lead to symptom re-occurrence and progression of the disease.

Up to 10% of WD is diagnosed in the presymptomatic stage from family screening. However, the main patterns of presentation of WD are hepatic or neurological symptoms or a combination of both [9, 10]. Up to 60% of patients present with hepatic symptoms at diagnosis and up to 100% have at least subclinical laboratory signs of liver damage over the disease course [11]. Neurological manifestations (mainly movement disorders) occur in 40–50% of patients at diagnosis. In addition to neurological symptoms, WD patients may have psychiatric symptoms (including affective, psychotic, behavioral, personality, anxiety and cognitive among others) before diagnosis, at diagnosis (10–25%) or during the course of the disease. Psychiatric complications more often occur in patients with neurological symptoms than other forms [11].

Very little is known about the social characteristics of patients with different forms of WD at diagnosis. The purpose of this cross-sectional study was to evaluate the social and demographic characteristics of patients with WD in Polish settings, particularly related to treatment persistence, which may influence socio-demographic parameters [8].

## Methods

The study was approved by the Bioethics Committee of the Institute of Psychiatry and Neurology in Warsaw, Poland and was conducted in line with the principles outlined in the Declaration of Helsinki.

The study included patients registered in the database of the Institute of Psychiatry and Neurology at Warsaw who were diagnosed with WD, started treatment between January 1995 and December 2005, and who were subsequently regularly treated or monitored at the Institute. The WD diagnosis was established based on the following criteria: history, physical examination, serum ceruloplasmin and copper levels, 24-h urinary copper excretion, and liver biopsy. In pre-symptomatic patients, the diagnosis was based on family screening and DNA analysis for known mutations. Predominant symptoms at diagnosis were classified into one of three forms: hepatic, neurological or asymptomatic as previously [12, 13].

Data were collected related to demographic characteristics, achieved education level, status of household, primary source of income, and marital status using a study specific questionnaire. From the remaining group, data were obtained by telephone contact. Responses given by the study subjects were verified, if possible, with information from the database and, if necessary, the medical history of each participant. Analyses based on level of education were conducted with a combined group of upper-secondary/post-secondary and higher education to reduce the chance that neurological patients upgrading their educational status would affect the results. Whenever possible, collected data were compared with Polish general population results obtained from the National Census of Population and Housing conducted by the Central Statistical Office in 2011 [14].

All data were analyzed in relation to predominant clinical form of disease (pre-symptomatic, hepatic or neurological) as well as by the level of persistence with medication use. Persistence with treatment was measured by the attending physician by detailed interview, analysis of refills, and drug consumption. Patients were considered non-persistent if they had at least one break of ≥3 months or more than 2 breaks lasting for ≥2 months within the last 3 years of treatment. On a random basis, and in all disputable cases, persistence was confirmed by tests of copper metabolism parameters: levels of serum copper and ceruloplasmin for patients treated with d-penicillamine and 24-h urine levels of zinc and copper for patients on zinc sulphate treatment.

Obtained data were collated in MS Excel and analyzed in two-tailed Fisher’s exact test, chi-square and unpaired Student’s t-test by the statistical software, Statistica.PL.

## Results

The study cohort comprised 202 subjects, 93 males (46%) and 109 females (54%). The demographic and clinical characteristics of subjects are presented in Table 1. Mean ± standard deviation age was 25.8 ± 9.8 years at diagnosis and 36.4 ± 9.9 years at assessment. The mean period of latency between first symptoms and diagnosis was 14.2 months. For the majority of patients, initial medication was d-penicillamine (57.1%), with zinc sulphate prescribed to 41.7%; treatment was initiated several days after diagnosis. The mean length of treatment was 11.0 years.

**Table 1:** Demographic and clinical characteristics of 202 subjects with Wilson disease included in the study population

Variable *n* = 202	Values
Male:female ratio, *n* (%)	93 (46):109 (54)
Age, mean ± SD (years)	36.4 ± 9.9
Age at diagnosis, mean ± SD (years)	25.8 ± 9.8
Latency period,^a^ mean ± SD (months)	14.2 ± 12.0
Length of treatment, mean ± SD (years)	11.0 ± 3.2
Predominant clinical form, *n* (%)	
- Pre-symptomatic	22 (10.9)
- Hepatic	73 (36.1)
- Neurological	107 (53.0)
Drug compliance (persistence),^b^ *n* (%)	
Yes	139^c^ (72.4)
No	53 (27.6)

*SD* standard deviation

^a^The period since first clinical symptoms

^b^Patients were considered non-persistent if they had at least one break of ≥3 months or more than 2 breaks lasting for ≥2 months within the last 3 years of treatment

^c^10 patients received liver transplants and stopped taking medication for Wilson disease

Social characteristics of study subjects versus the general population are presented in Table 2, with characteristics by predominant WD presentation in Table 3.

**Table 2:** Social characteristics of the study sample with Wilson disease and the Polish general population

Variable	Study sample *n* (%); *n* = 202	Polish general population [14], %
Education		
- Primary school	5 (2.5%)	1.4%
- Vocational education	51 (25.3%)	25.5%
- Upper-secondary/post-secondary and higher education	144 (62.9%)	73.1%
- Missing data	2 (1.0%)	–
Source of income		
- Salary	67 (33.2%)	37.8%
- Disability pension	76 (37.6%)	NA
- Pension (retired)	2 (1.0%)	27.4%
- Social support	11 (5.5%)	NA
- Other (self-employed, farming, student stipend)	31 (15.4%)	6.9%
- Family support	15 (7.4%)	27.9%
Status of household		
- Living alone	12 (5.9%)	NA
- With parents/guardians	48 (23.8%)	NA
- With wife/husband/informal relationship	59 (29.2%)	NA
- With family (wife/husband and children)	70 (34.7%)	NA
- Missing data	13 (6.4%)	NA
Marital status		
- Single	57 (28.2%)	27.2%
- Divorced	11 (5.5%)	4.4%
- Widowed	4 (2.0%)	9.4%
- Married	95 (47.0%)	54.6%
- Informal relationship	19 (9.4%)	3.0%
- Missing data	16 (7.9%)	1.4%

*NA* not available

**Table 3:** Social characteristics of the study sample with Wilson disease in relation to the predominant clinical form of the disease

Variable	Pre-symptomatic *n* = 22 *n* (%)	Hepatic *n* = 73 *n* (%)	Neurological *n* = 107 *n* (%)	*p*-value
Education				
- Primary school	1 (4.5)	1 (1.4)	3 (2.8)	NS
- Vocational	7 (31.8)	11 (15.1)	33 (30.8)	0.01
- Upper-secondary/post-secondary and higher education	13 (59.1)	61 (83.6)	70 (65.5)	0.003
- Missing data	1 (4.5)	0	1 (0.9)	NS
Source of income				
- Salary	5 (22.7)	41 (56.2)	21 (19.6)	< 0.0001
- Disability pension	0	19 (26.0)	57 (53.3)	0.0003
- Pension (retired)	0	0	2 (1.9)	NS
- Social support	0	2 (2.7)	9 (8.4)	NS
- Other (self-employed, farming, student stipend)	17 (77.3)	5 (6.9)	9 (8.4)	< 0.0001
- Family support	0	6 (8.2)	9 (8.4)	NS
Status of household				
- Living alone	2 (9.1)	4 (5.5)	6 (5.6)	NS
- With parents/guardians	13 (59.1)	17 (23.3)	18 (16.8)	0.0001
- With wife/husband/informal relationship	5 (22.7)	26 (35.6)	28 (26.2)	NS
- With family (wife/husband and children)	2 (9.1)	22 (30.1)	46 (43.0)	0.006
- Missing data	0	4 (5.5)	9 (8.4)	NS
Marital status				
- Single	14 (63.6)	21 (28.8)	22 (20.6)	0.0002
- Divorced	0	2 (2.7)	9 (8.4)	NS
- Widowed	0	1 (1.4)	3 (2.8)	NS
- Married	5 (22.7)	34 (46.6)	56 (52.3)	0.04
- Informal relationship	2 (9.1)	9 (12.3)	8 (7.5)	NS
- Missing data	1 (4.5)	6 (8.2)	9 (8.4)	NS

*NS* not significant

### Education

Obtained data suggest that WD has a somewhat negative impact on the achieved level of education: 62.9% of study subjects with WD completed upper-secondary/post-secondary or higher education compared with 73.1% of the general Polish population (Table 2, results NS). There were significant differences in relation to predominant clinical form of the disease (Table 3) – patients with neurological manifestations of WD less often achieved upper-secondary/post-secondary or higher education (combined) compared with those with hepatic manifestations (65.5% vs. 83.6%; *p* = 0.003). Pre-symptomatic patients could not be considered for comparison here as they were often much younger, with many still pursuing education.

### Source of income

Obtained data also showed that WD influences the ability to work: 37.6% of responders were receiving disability pension and, for 5.5%, the primary source of income was social support (Table 2). For comparison, in the general Polish population all non-labour related income groups totalled 27.4% only (*p* = 0.05). Again, highest differences were noticed based on primary presentation: only 19.6% of patients with neurological symptoms stated that a salary was their main source of income, while, considerably more, 56.2% of patients with hepatic presentation obtained a salary (*p* < 0.0001). In line with these findings, 53.3% of patients presenting neurological symptoms received a disability pension versus 26.0% of patients with hepatic presentation (*p* = 0.0003).

### Marital and household status

WD did not appear to form a barrier to finding a partner, getting married, and having children. Overall, 47.0% were married, 5.5% divorced, and 2.0% widowed, with 34.7% living with their spouses and children (Table 2). The percentage of married patients with WD was lower than in the general population (47.0% vs. 54.6%), although the results were not statistically significant (*p* = 0.2). No substantial differences between patients with neurological and hepatic forms of disease were noted regarding being married (52.3 and 46.6%, respectively) (Table 3).

Consistent with the younger age of pre-symptomatic WD patients (22.2 ± 4.2 years), they were more likely to be single (63.6%) or live with parents/guardians (59.1%) than those with neurological presentation (20.6 and 16.8%, respectively) or hepatic (28.8 and 23.3%, respectively; both results *p* < 0.001). Pre-symptomatic WD patients were also less likely to live with spouses and children (9.1%) than those with neurological presentation (43.0%) and hepatic presentation (30.1%; *p* = 0.006).

### Persistence with anticopper therapy

Of those receiving drug therapy, 53 patients (27.6%) were not persistent with treatment (Table 1). The worst persistence to drug treatment was noticed in the group of pre-symptomatic patients where 12 out of 22 subjects (54.6%) did not take medication consistently. There was no significant difference in non-persistence between those with neurological or hepatic presentation (24.7 and 21.4%, respectively).

There were no substantial differences in social and demographic characteristics with respect to prescribed pharmacotherapy (mainly d-penicillamine and zinc sulphate) (data not shown). However, there were significant differences depending on treatment persistence. Patients that continued drug treatment without breaks more frequently had upper-secondary, post-secondary, and higher education in comparison to those who were non-persistent (76.3% vs. 66.0%, *p* = 0.02) (Table 4). In addition, those who were persistent with treatment most often cited a salary as their primary income source than non-persistent patients (40.3% vs. 18.9%, *p* < 0.001).

**Table 4:** Social characteristics of the study sample^a^ with Wilson disease in relation to persistence with treatment

Variable	Persistent *n* = 139, % = 72.4 *n* (%)	Non-persistent *n* = 53, % = 27.6 *n* (%)	*p*-value
Education			
- Primary school	4 (2.9)	1 (1.9)	NS
- Vocational education	29 (20.9)	17 (32.1)	NS
- Upper-secondary/post-secondary and higher education	106 (76.3)	35 (66)	0.02
Source of income			
- Salary	56 (40.3)	10 (18.9)	< 0.001
- Disability pension	49 (35.3)	22 (41.5)	NS
- Pension (retired)	2 (1.4)	0 (0)	NS
- Social support	6 (4.3)	3 (5.7)	NS
- Other (self-employed, farming, student stipend)	18 (13.0)	11 (20.8)	NS
- Family support	8 (5.8)	7 (13.2)	NS
Status of household			
- Living alone	8 (5.8)	4 (7.6)	NS
- With parents/guardians	29 (20.9)	15 (28.3)	NS
- With wife/husband/informal relationship	44 (31.7)	14 (26.4)	NS
- With family (wife/husband and children)	50 (36.0)	17 (32.1)	NS
- Missing data	8 (5.8)	3 (5.7)	NS
Marital status			
- Single	39 (28.1)	16 (30.2)	NS
- Divorced	9 (6.5)	2 (3.8)	NS
- Widowed	3 (2.2)	1 (1.9)	NS
- Married	70 (50.4)	21 (39.6)	NS
- Informal relationship	11 (7.9)	7 (13.2)	NS
- Missing data	7 (5.0)	6 (11.3)	NS

*NS* not significant

^a^Only 192 patients continued pharmacotherapy, 10 patients had liver transplants and subsequently stopped taking medication prescribed for Wilson disease

## Discussion

Overall, WD appeared to have a negative impact on achieved level of education and influenced the ability to work as compared with the general Polish population. This negative impact was particularly observed in patients with predominantly neurological manifestations of the disease. Completion of upper-secondary/post-secondary or higher education was lower by patients with neurological manifestations than hepatic manifestations. We have also shown that patients with neurological symptoms were less likely to state a salary as their main source of income, compared with patients with hepatic manifestations, and more commonly received a disability pension.

WD starts early in life, around the time of secondary/higher education with a typical onset of around 15 to 30 years of age [5]. In the present study, mean age at diagnosis was 25.8 years and the mean latency period between appearance of initial symptoms and diagnosis/start of therapy was 14 months. The latency period is often shorter in patients with neurological symptoms than hepatic symptoms. Due to the pathogenesis of WD, hepatic manifestations start earlier than neurological manifestations; however, hepatic symptoms alone are often not recognised as WD and therefore the latency is later than with neurological symptoms.

Poorer functioning of neurological patients can be due to disability but may also be due to psychiatric/behavioral problems. It is common in WD to observe classic psychiatric syndromes in early adulthood, including behavioral and personality changes, anxiety, depression, manic and hypomanic syndrome, cognitive deficits, sleep problems, and sexual dysfunctions [11], which may all hinder the capacity to study and work**.** Behavioral and personality disorders are frequent psychiatric symptoms of WD, which are reported to occur in 46–71% of WD patients, with the most common manifestations of irritability, aggression, and antisocial behavior [11, 15, 16].

Despite the widespread development of psychiatric manifestations in WD, there are currently no treatment guidelines. Treatment of the psychiatric symptoms of WD is often guided by general psychiatric experience, which typically glosses over the specificity of WD [11]. Inadequate treatment of WD leads to deterioration of symptoms [17–21], which may prevent patients from being able to carry out full-time employment and make them dependent on governmental (disability and/or social) or family support.

Although not extensively studied in WD, the disease appears to affect health-related quality of life (HRQoL) [21]. Using the generic Short-Form 36-Item Health Survey (SF-36), lower scores were found in patients with neurological compared with those with a predominantly hepatic form of WD and HRQoL of patients with WD and psychiatric symptoms was also lower than that of those without them [21].

The percentage of married patients with WD was lower than in the general population, although the results were not statistically significant. One can envisage that the neurological manifestations may be a barrier to marriage; however, no substantial differences between patients with hepatic and neurological form of disease were noted regarding the proportion who were married. A follow-up study of the patients in later life could provide valuable insight of the characteristics over the course of the disease and in older patients with WD. The mean age of patients in our study was 36 years and it seems likely that the symptoms of WD, if not adequately treated, will have an even greater impact in older patients.

Of those receiving pharmacotherapy, over one-quarter were not persistent with treatment. Those who were persistent most often had upper-secondary, post-secondary, and higher education in comparison to those who were non-persistent, but we do not know whether treatment persistence determined education or education determined persistence. In addition, those who were persistent with treatment most often cited a salary as their primary income source than non-persistent patients. The cost of drugs was not a barrier for continuous treatment.

The results of the current study are consistent with our previous study where 25% of patients were not persistent [8]. In that study, persistence was associated with improvement in well-being as measured by the EQ-5D visual analogue scale [8]. In the present study, the worst treatment persistence was noted in the group of pre-symptomatic patients where over half did not take medication persistently. These data are also consistent with another of our studies in clinically pre-symptomatic patients, where 45% of patients were not persistent for at least 3 consecutive months and one-third were not persistent for more than 12 months [22]. Notably, persistent patients had a significantly higher likelihood of remaining symptom-free and their overall survival was similar to the survival rate observed in the general population. In the current study, pre-symptomatic patients were substantially younger than other groups and many still pursued their education. In several chronic asymptomatic diseases, patients frequently stop taking their medications because they don’t believe they need medication, consider them ineffective, or because they experience unpleasant side effects [23]. Indeed, current treatments for WD are subject to troublesome and potentially serious adverse effects, which need to be monitored [1].

The primary limitation of our study is based on the self-completing questionnaire. Psychopathological symptoms themselves may lead patients to give unreliable responses, although every effort was made to verify answers from the Institute’s database and medical records. We do not have detailed psychiatric status of patients at diagnosis and during treatment as they were managed by local psychiatrists.

## Conclusion

Neurological manifestations of WD and non-persistence with treatment had strong adverse effects on education level and work ability. Optimal treatment of asymptomatic and symptomatic patients, including emphasis and monitoring of treatment persistence, may help to reduce the impact of the disease on their normal lives.

## Data Availability

The datasets used and/or analyzed during the current study are available from the corresponding author on reasonable request.

## Abbreviations

HRQoL: Health-related quality of life
NA: Not available
NS: Not significant
SD: Standard deviation
WD: Wilson disease
